# Assessing the impact of the 2018 tetanus guidelines on knowledge and practices of emergency physicians in trauma patients: a national survey study

**DOI:** 10.7717/peerj.16032

**Published:** 2023-09-04

**Authors:** Junling Gao, Xiaxia Yu, Guanghui Cao, Xiaoming He, Pingde Zhang, Joseph Walline, Yuanxi Wang, Xingjuan Yu, Jun Xu, Thuan-Quoc Thach, Yong Liu

**Affiliations:** 1 Buddhist Practices and Counselling Science Lab, Centre of Buddhist Studies, The University of Hong Kong, Hong Kong SAR, China; 2 Department of Intensive Care Unit, Southern Medical University, Shenzhen, China; 3 School of Biomedical Engineering, Health Science Center, Shenzhen University, Shenzhen, China; 4 Emergency Department, State Key Laboratory of Complex Severe and Rare Diseases, Chinese Academy of Medical Science and Peking Union Medical College, Beijing, China; 5 Department of Neurology, Xiangyang Central Hospital, Hubei University of Arts and Science, Xiangyang, China; 6 Department of Surgery, The University of Hong Kong, Hong Kong SAR, China; 7 Accident and Emergency Medicine Academic Unit, The Chinese University of Hong Kong, Hong Kong SAR, China; 8 Department of Cardiac and Vascular Interventional Surgery, Shenzhen Hospital, Southern Medical University, Shenzhen, China; 9 Department of Traditional Chinese Medicine, Shandong College of Traditional Chinese Medicine, Yantai, China; 10 Department of Psychiatry, The University of Hong Kong, Hong Kong SAR, China

**Keywords:** Chinese Expert Consensus Guidelines, CECG, Knowledge, attitudes, and practices (KAP), Tetanus toxoid, Tetanus

## Abstract

**Background:**

Tetanus remains a significant public health issue in China, with the approach of anti-tetanus prophylaxis in the emergency department resulting in both overuse, particularly of human tetanus immune globulin (TIG), and underuse with the tetanus vaccine. This is largely due to the absence of updated guidelines on tetanus prophylaxis before 2018. Our study aimed to evaluate the effects of the 2018 Chinese tetanus guidelines on the knowledge and practices of emergency physicians about tetanus prevention in trauma patients.

**Methods:**

From November 2019 to April 2020, we conducted a web-based survey involving 499 emergency physicians. The survey included a questionnaire covering knowledge, attitudes, and practices related to tetanus. We assessed the influence of the 2018 tetanus guidelines on the knowledge and practices of emergency physicians related to tetanus prevention for patients with trauma using multiple regression analysis.

**Results:**

The survey results showed that only 45.3% of the participants had received formal training on tetanus immunization, despite 53.3% reporting the availability of tetanus vaccines at their institutions. Physicians typically prescribed tetanus antitoxin or human TIG instead of tetanus toxoid (TT) to treat injuries, regardless of the patient’s TT vaccination history. Among the respondents, those who were aware of the 2018 tetanus guidelines had higher mean scores on the general knowledge, risk knowledge, and treatment knowledge scales, with increases of 6%, 13%, and 9%, respectively, compared to those who were unaware of the guidelines. Awareness of the 2018 tetanus guidelines was associated with a high level of knowledge, as indicated by the general knowledge score, recommendation knowledge score, and total knowledge score, after adjusting for the effects of all variables on the knowledge, attitudes, and practices of the participants. A high level of education was also associated with a high level of knowledge indicated by the recommendation knowledge score and total knowledge score.

**Conclusions:**

Our study highlights a substantial gap in the attitudes, knowledge, and practices of emergency physicians in China regarding tetanus immunization. The results suggest an urgent need to promote the Chinese Expert Consensus Guidelines on tetanus to improve emergency physicians’ knowledge and competence in tetanus prophylaxis. The findings underscore the importance of enhancing physicians’ awareness of the latest guidelines to ensure appropriate and effective treatment for patients with tetanus-prone injuries.

## Introduction

Tetanus is a rare and potentially fatal infectious disease with a mortality rate ranging from 10% to 80% (Finkelstein et al., 2017). The disease is preventable by vaccination and post-exposure prophylaxis (Thwaites & Loan, 2015). Despite a century of efforts to control the disease through routine childhood tetanus vaccination and high childhood immunization coverage over the past 50 years, tetanus remains a pandemic. China has eliminated maternal and neonatal tetanus by strengthening routine immunization and reproductive health services (Zheng et al., 2018). To improve healthcare for newborns and children aged below 14 years, the Chinese Government made the most common vaccinations, such as diphtheria, tetanus, and pertussis (DTP), mandatory and easily accessible. However, the tetanus vaccination status of adults in China is often overlooked (ASHP, 2022; Zheng et al., 2018). This may be due to the lack of updated guidelines on tetanus prophylaxis until 2018. The anti-tetanus prophylaxis currently used in emergency departments has led to overtreatment. Indeed, human tetanus immune globulin (TIG) or tetanus antitoxin (TAT) is overused for tetanus post-exposure prophylaxis in patients with clean, minor wounds (regardless of their immunization status) or for patients with tetanus-prone wounds who have previously received ≥3 doses of a preparation containing tetanus toxoid adsorbed, particularly with TIG, and under-treatment with the tetanus vaccine, which is not properly used for tetanus prophylaxis when indicated by ACIP or similar guidelines (Wang et al., 2019). In comparison, vaccination is mandatory for infants and toddlers aged 0 to 24 months (Zhang et al., 2022b). The vaccination coverage rate (VCR) among adults aged over 15 years was low and decreased with age (Gergen et al., 1995; Marchal et al., 2021), especially in China (Huang, Xu & Yang, 2009; Liu, Lu & Shan, 2009).

There is a lack of epidemiological data on non-neonatal tetanus in China, but in some reports, certain hospitals admitted more than 30 tetanus patients in a year (Gou et al., 2023). Furthermore, seroprevalence data found that low antibody levels are common in young adults and antibody levels tend to decrease with age (Nazik et al., 2020; Zhang et al., 2022a). The data suggest that poor compliance with booster recommendations may contribute to a relatively high incidence of tetanus, in contrast to China’s economic growth. The guidelines of nearly all developed countries recommend that primary care and emergency clinics provide pre- and post-exposure tetanus immunization to non-immunized individuals, along with booster shots to previously immunized adults (Havers et al., 2020; Hibberd, 2023; Lisboa et al., 2011; Committee on Infectious Diseases, 2006; World Health Organization (WHO), 2023). Five doses are recommended during childhood, with a sixth given during adolescence. Subsequently, additional doses are recommended every 10 years or after exposure.

However, the Chinese National Immunization Program only recommends providing free tetanus immunization to children under 7 years old but offers no guidelines concerning active immunization among adults. As a result, a majority of adolescents and adults do not receive further immunization boosters for about 5 to 10 years after completion of the childhood tetanus vaccination, and thus, remain unprotected and susceptible to tetanus (Wang, 2019). It was not until 2018 that the Chinese Expert Consensus Guidelines (CECG) began to promote the prevention and management of accidental tetanus in adult patients (Wang et al., 2019). Health workers’ knowledge, attitudes, and practices (KAP) are critical for ensuring general awareness of vaccination procedures and their effectiveness, especially in the case of the tetanus vaccine (Riccò et al., 2017a, 2017b). Updated KAP can universally raise general awareness and promote vaccination status in the population by following proper guidelines (Betsch & Wicker, 2014; Kirupakaran, Meloche & Upfal, 2018). It is purported that outdated knowledge, loose attitudes, or improper practices regarding the prevention and treatment of tetanus are not uncommon among physicians, and each factor negatively influences the vaccination status of populations (Zaki et al., 2018).

We previously conducted a pilot study on assessing the effectiveness of such Guidelines with a sample size of 197 emergency physicians (Liu et al., 2020). From November 2015 to April 2016, we surveyed physicians’ knowledge and practices on adults’ tetanus vaccination in China. The sample size of the study was small, and the representation of respondents was limited to some parts of China. The study was conducted before the publication of the CECG on the prevention and management of accidental tetanus in adult patients in December 2018 and could not assess the impact of the guideline on KAP among emergency physicians.

To date, few studies on emergency physicians’ KAP regarding tetanus vaccination have been conducted in Mainland China, and little is known about emergency physicians’ tetanus-related knowledge and practices since the publication of new consensus guidelines in 2018 (Wang et al., 2019). This consensus is optional but not mandatory, so it’s unclear whether emergency physicians adhere to this consensus and how it affects their KAP. Therefore, we hypothesized that the publication of this guideline will improve the knowledge of Chinese emergency physicians, change the attitude towards active immunization, and recognize the misconception of TIG overuse. This study aimed to identify factors influencing KAP for tetanus vaccination and common knowledge among emergency physicians in Mainland China. In addition, we investigated whether the recently published consensus guidelines for tetanus vaccination have an impact on KAP.

## Materials and Methods

We conducted a web-based survey of emergency physicians in Mainland China to evaluate their KAP related to tetanus vaccination in trauma patients, in light of the recently published consensus guidelines on this topic. The study was approved by the Ethics Committee of the Shenzhen Hospital of Southern Medical University (SZYYEC-201804-K6).

### Data collection, sample size calculation, and sampling

We used a convenience sampling method to recruit participants for the survey, which was conducted from November 2019 to April 2020. Recruitment was done through advertisements and WeChat invitations sent *via* the Emergency Medicine WeChat subscription account, one of the largest WeChat accounts in China. Emergency physicians were selected as the primary respondents for this study, as the emergency department is the primary site of tetanus prophylaxis for trauma patients. The questionnaires were administered through a leading online survey website. To determine the sample size, we used the formula *n* = Z_α/2_ × p × (1-p)/ε², where Z_α/2_ represents the value of the standard normal distribution, ε denotes the margin of error, and p represents the prevalence of tetanus. Assuming α = 0.05, then Z_α/2_ = 1.96 with a margin error of 5%, a minimum of 385 participants were required. As the prevalence of tetanus in China was unknown, we assumed the prevalence *p* = 0.05 to estimate the sample size (Kohn & Senyak, 2021).

### Questionnaires

We used a pre-designed, validated questionnaire adapted from the 2018 China tetanus guidelines to assess the knowledge, attitudes, and practices of emergency physicians regarding tetanus vaccination in trauma patients. The questionnaire comprised 35 closed-ended questions, grouped into four categories of information. The categories included basic information on participating physicians (Questions 1–7), information on tetanus immunization practices implemented by physicians and their institutions (Questions 8–10, 13, and 22), physicians’ general knowledge of tetanus (Questions 11, 12, and 14–21), physicians’ attitudes toward tetanus immunization (Questions 23–27), and physicians’ knowledge of the CECG on trauma patients’ tetanus vaccination (Questions 28–35). The questionnaire was designed to be comprehensive, covering various aspects of tetanus immunization, and was adapted from the 2018 China tetanus guidelines.

We scored physicians’ knowledge and practices based on the sum of correct responses to relevant survey questions, with a response defined as correct if it agreed with the new consensus guidelines on tetanus vaccination. Unanswered questions were scored as incorrect (Table S1). Before data collection, we conducted a pre-test of the questionnaire among 5% of the sample size, comprising emergency physicians in Shenzhen city, and made necessary adjustments based on feedback received. During data collection, supervisors checked the collected data daily to ensure completeness. To ensure the reliability and validity of the questionnaire, we calculated Cronbach’s alpha, resulting in a value of 0.70 for the total knowledge score and 0.62 for the recommendation knowledge score, indicating moderate reliability. We also recruited experts to evaluate the questionnaire’s content using face validity, and they agreed that the questionnaire was a valid measure of the concept being measured, indicating that the measured items matched the given domain of the concept.

### Attitudes

Respondents used a five-point Likert scale to rate their specific attitudes towards statements about TAT, TIG, and vaccines. To calculate the accumulated vaccine propensity score, we added points corresponding to each attitude. For each propensity rated “strongly disagree,” one point was added, two points were added for “disagree,” and so on. The cumulative scores for TAT or TIG were referred to as the attitude TAT or TIG score. We converted the cumulative scores referred to as vaccine scores into percentages.

In this article, we define the term “favourable” as a situation where at least 80% of respondents have selected a particular option in the questionnaire. We also use the term “adequate knowledge” to refer to a question’s accuracy rate of over 60%. An “optimal” attitude toward TIG or TAT is defined as a proportional score of less than 60%, while an “optimal” attitude toward vaccines is defined as a proportional score of more than 80%. We refer to a situation where less than 60% of respondents have selected a particular option in the questionnaire as a “suboptimal” attitude, knowledge, or practice.

### General knowledge and knowledge of official recommendations

The survey included a general knowledge test with 10 multiple-choice questions covering common misconceptions regarding vaccination and physicians’ general knowledge regarding TAT. Participants also received questions with respect to official consensus recommendations on tetanus vaccination. We calculated the accumulative general knowledge score (GKS) and the recommendation knowledge score (RKS) related to tetanus vaccination by adding one point to the participant’s score for each correct answer. The total knowledge score (TKS) is the sum of the GKS and RKS. All cumulative scores were converted to percentages.

We define a “high” corresponding propensity score as an answer with an accuracy rate of over 80%. Conversely, we refer to an answer with an accuracy rate of less than 60% as indicating “poor” knowledge.

### Statistical analysis

We performed data analysis using R version 4.0.3 (R Core Team, 2020). Continuous variables were presented as mean ± standard deviation and tested for normal distribution using the Kolmogorov-Smirnov test. We used the Student’s t-test to compare the mean values of normally distributed continuous variables and the Mann-Whitney U test for non-normally distributed data. Categorical variables were expressed as percentages and compared using Pearson’s Chi-squared test. Independent variables included gender, age, education level, information resource, and practice region. We assessed associations between independent variables and the scores of attitudes (attitude score) and knowledge (GKS, RKS, and TKS), particularly the impacts of the 2018 guidelines on the knowledge and practices of tetanus prevention among emergency physicians in trauma patients. In multiple regression analyses, we used a cut-off of *p* < 0.25 for the inclusion of independent variables in the model. The multiple linear regression model used a backward elimination procedure in which the least significant predictor in the model was eliminated sequentially. At each step, we checked estimates to ensure that dropping the least significant variable did not affect other variables. The final model retained only those factors that were significant at *p* < 0.05. We considered a two-tailed *p* < 0.05 to be statistically significant.

## Results

**Figure 1:**
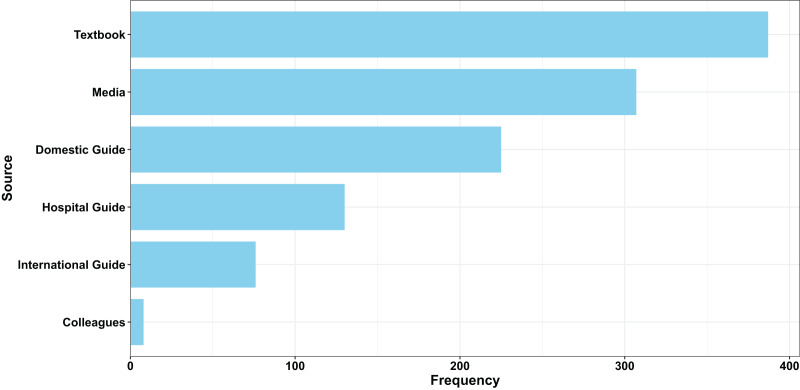
Collected physicians’ information from multiple sources.

A total of 499 questionnaires were analyzed, resulting in a response rate of 81.8% (499/610). Table 1 presents the general characteristics of the respondents. The majority of respondents (85%) were male, with over 46% aged between 30 and 40 years. After stratifying the country by administrative subregions, 14.6% were from Eastern China, 15.8% from Western China, 16.6% from Central China, 28.7% from Northern China, and 21.8% from Southern China. Of the 499 respondents, 320 (64.1%) had a 5-year college degree, and 169 (33.9%) possessed a postgraduate degree. Table 2 shows that only 45.3% of physicians received formal training on tetanus vaccination, and most of them were not aware of the standard tetanus vaccination schedules. Additionally, only 85.8% of respondents received a tetanus vaccine booster in the past 10 years, and 53.3% confirmed the availability of the tetanus vaccine in their respective institutions. Respondents primarily learned about tetanus from textbooks and colleagues (Fig. 1).

**Table 1:** Characteristics of participants of the survey (*N* = 499).

Characteristics	*N* (%)
**Age**	
<30	41 (8.2%)
30–40	232 (46.5%)
40–50	183 (36.7%)
≥50	43 (8.6%)
	
**Gender**	
Female	75 (15%)
Male	424 (85%)
	
**Geographical origin**	
Eastern China	73 (14.6%)
Western China	79 (15.8%)
Central China	83 (16.6%)
North China	143 (28.7%)
South China	109 (21.8%)
Overseas	12 (2.4%)
	
**Educational level**	
Undergraduate	320 (64.1%)
Postgraduate	169 (33.9%)
	
**Organization type**	
Primary hospital	14 (2.8%)
Secondary hospital	114 (22.7%)
Tertiary hospital	374 (74.5%)

**Table 2:** Counts and proportions of physicians receiving tetanus vaccination in different regions across mainland China (*N* = 499).

Characteristics	Number of cases	Northern China	Eastern China	South China	Western China	Central China	Overseas
Tetanus vaccination training	226 (45.3%)	59 (11.8%)	24 (4.8%)	48 (9.6%)	42 (8.4%)	46 (9.2%)	7 (1.4%)
Physicians receiving a tetanus booster in the past 10 years	428 (85.8%)	118 (23.6%)	67 (13.4%)	91 (18.2%)	68 (13.6%)	73 (14.6%)	11 (2.2%)
Supply of tetanus vaccine	266 (53.3%)	92 (18.4%)	28 (5.6%)	56 (11.2%)	45 (9.0%)	38 (7.6%)	7 (1.4%)

**Figure 2:**
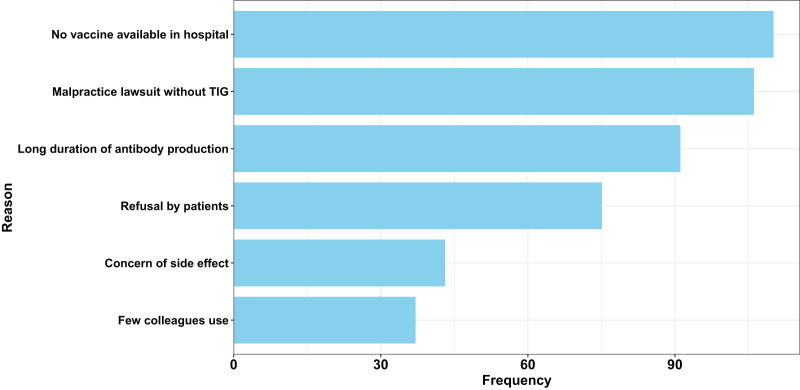
Attitude towards tetanus vaccination: main reasons given for acceptance of tetanus vaccine among all respondents. TIG, Tetanus immune globulin.

Table 3 explores attitudes towards tetanus vaccination and shows that the majority of participants highly favoured TAT and TIG. About 94% of participants favoured TAT, while 95.2% favoured TIG. These participants who favoured TAT (*n* = 469, 94%) or TIG (*n* = 475, 95.2%) had a high corresponding propensity score of 89.7 ± 16.4% for TAT and 88.7 ± 14.6% for TIG (actual range, 20.0–100%). Those who favoured vaccination (*n* = 434, 87.0%) also had a high corresponding propensity score of 91.8 ± 22.7%. There were no significant differences by gender, age, educational level, type of institution, or geographical region between those who favoured vaccination. The main reasons for choosing TIG for tetanus prophylaxis were the lack of tetanus vaccine at the hopital where they worked and the fear of litigation (Fig. 2).

**Table 3:** Distribution of knowledge and attitude scores for demographics and characteristics of participants (*N* = 499).

	Attitude towards TAT/TIG		Attitude towards vaccine		General knowledge		Recommendation vaccine		Total knowledge	
	Mean ± SD (%)	*P*-value	Mean ± SD (%)	*P*-value	Mean ± SD (%)	*P*-value	Mean ± SD (%)	*P*-value	Mean ± SD (%)	*P*-value
**Information sources**		**0.011**		**0.018**		**0.001**		**0.001**		**0.001**
Non-domestic guidelines	88.2 ± 13.3		92.3 ± 19.6		37.3 ± 13.8		27.6 ± 18.2		33.0 ± 11.8	
Domestic guidelines	89.2 ± 12.5		93.6 ± 17.7		43.4 ± 11.4		39.5 ± 19.5		41.7 ± 12.0	
										
**Age in years**		0.863		0.395		0.996		0.436		0.705
<30	91.2 ± 9.7		92.8 ± 17.2		40.4 ± 19.7		28.5 ± 14.7		35.1 ± 8.0	
30–40	89.2 ± 12.3		91.4 ± 20.6		40.2 ± 13.2		34.2 ± 18.9		37.5 ± 22.1	
40–50	89.1 ± 12.9		94.4 ± 16.7		39.9 ± 13.2		32.4 ± 21.2		36.6 ± 13.7	
≥50	88.6 ± 13.2		94.4 ± 15.9		39.8 ± 14.7		31.1 ± 19.9		35.9 ± 13.8	
										
**Gender**		0.931		0.342		0.270				
Female	89.3 ± 12.3		91.8 ± 20.2		41.6 ± 12.7		33.3 ± 18		37.9 ± 11.1	
Male	89.2 ± 12.5		93.0 ± 18.5		39.8 ± 13.2		32.9 ± 20		36.7 ± 12.9	
										
**Institution**		0.574		0.097		**0.023**		0.870		0.460
Clinic	91.4 ± 10.3		95.7 ± 16.0		36.0 ± 9.0		32.0 ± 17.0		34.0 ± 9.0	
Secondary hospital	87.8 ± 14.9		95.3 ± 15.9		38.0 ± 14.0		33.0 ± 21.0		36.0 ± 14.0	
Tertiary hospital	89.5 ± 11.8		92.0 ± 19.5		41.0 ± 13.0		33.0 ± 19.0		37.0 ± 12.0	
										
**Education level**						**0.040**		**0.020**		
Undergraduate	93.1 ± 18.4		93.1 ± 18.4		39.2 ± 13.1		31.5 ± 19.4		35.8 ± 12.6	
Postgraduate	92.4 ± 19.4		92.4 ± 19.4		41.8 ± 13.1		35.9 ± 20.2		39.2 ± 12.6	
										
**Area**		0.886		0.178		**0.001**		**0.002**		0.141
Northern	89.7 ± 12.1		95.7 ± 14.7		37.2 ± 12.5		35.0 ± 21.0		36.2 ± 13.1	
Eastern	88.5 ± 14.1		91.0 ± 20.8		41.5 ± 11.7		35.0 ± 22.0		38.6 ± 13.4	
Southern	87.7 ± 14.4		91.2 ± 20.3		42.5 ± 12.6		32.5 ± 19.1		38 ± 12.1	
Western	92.4 ± 8.1		93.2 ± 19.2		37.5 ± 15.2		29.1 ± 17.3		33.8 ± 12.3	
Central China	88.1 ± 11.8		91.8 ± 20.2		43.1 ± 12.4		31.9 ± 16.6		38.2 ± 1.07	
Overseas	88.3 ± 10.3		91.7 ± 19.9		39.2 ± 15.6		35.4 ± 27.6		37.5 ± 27.6	

**Note:**

TAT, Tetanus antitoxin; TIG, tetanus immune globulin; SD, standard deviation. Values in bold represent statistically significant results.

The survey results showed that respondents’ knowledge of tetanus vaccination was inadequate. After normalizing, the mean total knowledge score (TKS) was only 36.0 ± 12.7% (actual range, 0.0–100.0), and the mean general knowledge score (GKS) and recommendation knowledge score (RKS) were respectively 40.1 ± 13.1% and 30.0 ± 19.7%. The practices followed by most respondents significantly differed from the CECG on tetanus vaccination with low correct responses (the correct response percentages were 10.4%, 64.5%, 29.7%, 10.6%, 8.2%, and 6.6% for Questions 30–35, respectively). Furthermore, participants’ responses showed an overuse of TIG treatment, as a high proportion of participants who responded to questions with the option of TIG treatment only. Specifically, 58.7% of participants selected this option for Question 30, 29.3% for Question 31, 38.1% for Question 32, 63.7% for Question 33, 50.7% for Question 34, and 42.3% for Question 35. As shown in Table 3, mean scores of GKS, RKS, and TKS for participants with a postgraduate degree were significantly greater than those without a postgraduate degree, with means of 41.8 ± 13.1%, 35.9 ± 20.2%, and 39.2 ± 12.6%, respectively, compared to 39.2 ± 13.1%, 31.5 ± 19.4%, and 35.8 ± 12.6% for participants without a postgraduate degree (*P*-value = 0.01). These scores were higher in participants with information collected from different sources (the mean values of GKS, RKS, and TKS in participants who were aware of the expert consensus guidelines were 43.9 ± 19.1%, 43.8 ± 22.5%, and 43.8 ± 12.4%, respectively). Tables 3 and 4 reveal that there were no significant differences in scores by gender, geographical region, or age group. However, participants’ knowledge levels were moderately impacted by their awareness of the CECG. The mean scores of GKS, RKS, and TKS in participants who were aware of these guidelines were 6%, 13%, and 9%, respectively higher than those who were not. Even after adjusting for the interrelated effects of all other variables such as age, gender, education level, and geographical region, the scores for general knowledge, recommendation vaccine, and total knowledge were slightly higher for those who were aware of the CECG compared to those who were not aware (the regression coefficients for awareness of CECG for general knowledge score, recommendation vaccine score, and total score in participants were 0.055, 0.055, and 0.084, respectively). Additionally, education attainment also had an impact on the recommendation vaccine score and total knowledge score. The scores for recommendation vaccine and total knowledge were moderately higher for those who were aware of CECG than those who were not aware (39.5 ± 19.5% *vs* 27.6 ± 18.2% and 41.7 ± 12.0% *vs* 33.0 ± 11.8%, respectively). After adjusting for the interrelated effects of all variables on the KAP, the scores for recommendation vaccine and total knowledge for participants with a postgraduate degree were moderately higher than for those without (regression coefficients for participants who have completed a postgraduate degree were 0.029 and 0.037 for the recommendation vaccine score and total knowledge, respectively).

**Table 4:** Multiple linear regression for scores of knowledge and attitude for different demographics and characteristics of participants (*N* = 499).

Variable	Attitude towards TAT/TIG		Attitude towards vaccine		General knowledge		Recommendation vaccine		Total knowledge	
	Coefficient regression [95% CI]	*P*-value	Coefficient regression [95% CI]	*P*-value	Coefficient regression [95% CI]	*P*-value	Coefficient regression [95% CI]	*P*-value	Coefficient regression [95% CI]	*P*-value
**Age in years**										
<30	Ref	Ref	Ref	Ref	Ref	Ref	Ref	Ref	Ref	Ref
30–40	−0.229 [−0.752 to 0.294]	0.390	−0.117 [−0.509 to 0.275]	0.558	0.004 [−0.049 to 0.057]	0.884	0.043 [−0.036 to 0.121]	0.283	0.021 [−0.029 to 0.071)	0.402
40–50	−0.234 [−0.771 to 0.302]	0.391	0.005 [−0.397 to 0.407]	0.981	0.011 [−0.43 to 0.065]	0.690	0.022 [−0.059 to 0.102]	0.592	0.016 [−0.029 to 0.071]	0.541
≥50	−0.306 [−0.937 to 0.326]	0.342	0.016 [−0.457 to −0.490]	0.946	0.014 [−0.050 to 0.078]	0.667	0.027 [−0.068 to 0.122]	0.573	0.020 [−0.055 to 0.071]	0.517
**Gender**										
Female	Ref	Ref	Ref	Ref	Ref	Ref	Ref	Ref	Ref	Ref
Male	−0.015 [−0.326 to 0.297]	0.927	0.045 [−0.188 to 0.279]	0.703	−0.018 [−0.050 to 0.013]	0.257	0.003 [−0.044 to 0.050]	0.911	−0.009 [−0.039 to 0.019]	0.554
**Institution**										
Clinic	Ref	Ref	Ref	Ref	Ref	Ref	Ref	Ref	Ref	Ref
Secondary hospital	−0.356 [−1.057 to 0.345]	0.319	−0.019 [−0.545 to 0.506]	0.942	0.021 [0.050 to 0.092]	0.561	−0.012 [−0.118 to 0.093]	0.816	0.006 [−0.061 to 0.072]	0.857
Tertiary hospital	−0.196 [−0.881 to 0.489]	0.574	−0.206 [−0.719 to 0.308]	0.432	0.040 [−0.029 to 0.110]	0.253	−0.032 [−0.135 to 0.070]	0.535	0.008 [−0.057 to 0.073]	0.810
**Education level**										
Undergraduate	Ref	Ref	Ref	Ref	Ref	Ref	Ref	Ref	Ref	Ref
Postgraduate	0.011 [−0.256 to 0.278]	0.934	0.062 [−0.138 to 0.262]	0.546	0.013 [−0.014 to 0.040]	0.352	**0.045 [0.007 to 0.080]**	**0.029**	**0.027 [0.002–0.052]**	**0.037**
**Area**										
Northern	Ref	Ref	Ref	Ref	Ref	Ref	Ref	Ref	Ref	Ref
Eastern	−0.137 [−0.496 to 0.221]	0.452	−0.244 [−0.513 to 0.025]	0.075	0.040 [0.004 to 0.076]	0.031	−0.012 [−0.556 to 0.055]	0.660	0.017 [−0.017 to 0.051]	0.334
Southern	−0.198 [−0.516 to 0.120]	0.221	−0.214 [−0.453 to 0.024]	0.076	0.051 [0.019 to 0.083]	0.002	−0.034 [−0.074 to 0.024]	0.167	0.013 [−0.017 to 0.051]	0.388
Western	0.258 [−0.095 to 0.612]	0.152	−0.082 [−0.347 to 0.183]	0.543	0.004 [−0.031 to 0.040]	0.820	−0.050 [−0.113 to 0.004]	0.066	−0.020 [−0.053 to 0.014]	0.249
Central China	−0.189 [−0.537 to 0.160]	0.287	−0.192 [−0.453 to 0.069]	0.149	0.051 [0.015 to 0.086]	0.005	−0.053 [−0.084 to 0.023]	0.049	0.005 [−0.028 to 0.038]	0.774
Oversea	−0.126 [−0.875 to 0.623]	0.741	−0.233 [−0.795 to 0.329]	0.415	0.005 [–0.070 to 0.081]	0.889	0.013 [−0.126 to 0.099]	0.816	−0.003 [−0.074 to 0.068]	0.936
**Information**										
Non-domestic	Ref	Ref	Ref	Ref	Ref	Ref	Ref	Ref	Ref	Ref
Domestic	−0.158 [−0.382 to 0.065]	0.164	0.095 [−0.073 to 0.262]	0.267	**0.055 [0.033 to 0.078]**	**0.002**	**0.055 [0.087 to 1.154]**	**0.000**	**0.084 [0.063 to 0.105]**	**0.000**

**Note:**

TAT, tetanus antitoxin; TIG, tetanus immunoglobulins; CI, confidence interval. Values in bold represent significant results.

## Discussion

This cross-sectional study examined the KAP of Chinese emergency physicians concerning tetanus vaccination. We uncovered the following key findings on emergency physicians’ KAP regarding tetanus vaccination for trauma patients in Mainland China:

1. Tetanus immune globulin is overused as a treatment for trauma patients rather than tetanus antitoxin.

2. Most emergency physicians had a favourable attitude towards TIG and TAT for tetanus immunization.

3. A majority of emergency physicians were not adequately vaccinated, and the tetanus vaccine was available only at a small number of respondents’ institutions.

4. Physicians’ knowledge of tetanus treatment was generally poor, with higher knowledge levels among those informed of the latest published consensus guidelines on tetanus vaccination in China and those who received higher education.

Emergency clinicians generally had a favourable attitude towards TAT, TIG, and TT vaccination. Sociodemographic characteristics, such as gender, education level, and the geographical region where physicians practised, did not affect their attitude towards TIG or vaccination, nor their knowledge level regarding tetanus vaccination. The preference for TAT and TIG is consistent with our previous research. The Chinese guidelines indicate that prophylaxis with TIG is a routine practice among Chinese emergency physicians, a finding not found in other countries. Our study suggests that this discrepancy may be due to multiple factors, including the vaccine distribution system in China, concerns over malpractice lawsuits, and misconceptions about the long-term antibody response induced by the toxoid.

We found that the recently published CECG on tetanus vaccination significantly influenced physicians’ knowledge levels. However, only 15.4% of physicians reported updating their knowledge by reading these guidelines, a relatively low percentage considering their recent publication. Physicians’ knowledge levels were moderately impacted by familiarity with the Chinese expert consensus. Despite criticisms of TIG and TAT overuse in Chinese medical literature, they remain the primary tetanus prophylaxis treatments used by Chinese emergency physicians. However, the CECG’s publication resulted in a modest positive influence on physicians’ knowledge and practices, warranting further promotion among medical professionals.

Controversial outcomes were found with respect to the use of the TT vaccination booster. Although China eliminated maternal and neonatal tetanus by 2012 without using the vaccine as other countries typically have, this success is primarily due to improved birth hygiene and increased in-hospital delivery rates. These results may lead some to believe TT vaccination boosters are unnecessary, but adult tetanus cases have been frequently reported in Chinese literature (Gou et al., 2023). Tetanus antibody levels tend to decrease with age, putting adults over 20 years old at high risk of tetanus infection. This suggests that adults over 20 years old should receive one TT dose as a booster (Liu, Lu & Shan, 2009; Marchal et al., 2021).

Our survey demonstrated that TAT and TIG overuse is prevalent in China, with 30–50% of participants choosing TAT or TIG as the preferred post-exposure prevention treatment for patients who had completed a three-dose primary series. Possible reasons for this include recommendations in Chinese textbooks, lack of understanding among physicians regarding tetanus pathogenesis, and misconceptions about TAT and TIG as gold standards for tetanus prophylaxis. Side effects stemming from TAT and TIG overuse in China have been widely reported and should no longer be overlooked (Zheng et al., 2018).

Additionally, Savage et al. (2007) and Donken et al. (2014) reported their findings on pool adherence that is consistent with our tetanus prophylaxis recommendations. They found that over-immunised patients should not be vaccinated due to the uncertainty of the vaccination history. The respondents in their study tend to prescribe vaccines instead of TAT or TIG.

Despite increased awareness of over- and undertreatment with tetanus immunoglobulin and vaccine, the knowledge, attitudes, and practices of emergency physicians in China regarding tetanus immunization remain suboptimal. To address this issue, we recommend establishing regular stakeholder audit mechanisms, defining a core set of quality measures, and improving vaccine availability. Although our study has limitations, it underlines the urgent need for promoting Continuing Education Credit Grants (CECGs) on tetanus among physicians in China, to update their knowledge and competencies regarding tetanus immunization.

Our study has several limitations, including unverified vaccination certificates and physician licenses, a cross-sectional survey design, non-probabilistic sampling, and a homogeneous target population limited to emergency physicians in Mainland China.

## Conclusions

Our study highlights a substantial gap in the attitudes, knowledge, and practices of emergency physicians in China regarding tetanus immunization. The results suggest an urgent need to promote the Chinese Expert Consensus Guidelines on tetanus to improve emergency physicians’ knowledge and competence in tetanus prophylaxis. The findings underline the importance of enhancing physicians’ awareness of the latest guidelines to ensure appropriate and effective treatment for patients with tetanus-prone injuries.

## Supplemental Information

10.7717/peerj.16032/supp-1 Supplemental Information 1 Data.

10.7717/peerj.16032/supp-2 Supplemental Information 2 Knowledge test of items proposed to the 707 Agricultural Workers from the Autonomous of Trento participating in the survey.

10.7717/peerj.16032/supp-3 Supplemental Information 3 Questionnaire in Chinese.

10.7717/peerj.16032/supp-4 Supplemental Information 4 Questionnaire in English.

## Additional Information and Declarations

### Data Availability

The following information was supplied regarding data availability: The raw data is available in the Supplemental File.
