# Supplementary material for: Assessing the impact of the 2018 tetanus guidelines on knowledge and practices of emergency physicians in trauma patients: a national survey study
Source: PeerJ. 2023 Sep 4;11:e16032. doi: 10.7717/peerj.16032 (PMC10484204; doi:10.7717/peerj.16032)
Supplement: Supplemental Information 2 [file peerj-11-16032-s002.docx]

| **Type** | **Question no** | **Questions / Statements** |
| --- | --- | --- |
| GSK | 11 | Are tetanus antitoxin or human TIG (human tetanus immunoglobulin) antibodies or antigens? |
|  | 12 | Are tetanus-containing vaccines (DTaP, Tdap, DT, Td, or TT) antibodies or antigens? |
|  | 14 | How long is the incubation period of tetanus after trauma? |
|  | 15 | What is the half-life of tetanus antitoxin? |
|  | 16 | How many injections does an unvaccinated patient need to get a protective antibody titer? |
|  | 17 | The appropriate time to implement preventive measures against tetanus after trauma is: |
|  | 18 | Do patients with intestinal perforation need to take preventive measures against tetanus? |
|  | 19 | Do pregnant women need to take preventive measures against tetanus? |
|  | 20 | Do adults who have completed the national immunization program in childhood need to be vaccinated against tetanus? |
|  | 21 | Which of the following statements is true about TAT or TIG? |
| RSK | 28 | The recommended use of tetanus vaccine for trauma patients with no history of tetanus vaccination is: |
|  | 29 | The recommended use of tetanus vaccine for trauma patients with incomplete 3-dose primary series with any tetanus-containing vaccine |
|  | 30 | The recommended use of tetanus vaccine for trauma patients with clean and minor wounds and incomplete 3-dose primary series with any tetanus-containing vaccine |
|  | 31 | The recommended use of tetanus vaccine for trauma patients with dirty and major wounds and incomplete 3-dose primary series with any tetanus-containing vaccine |
|  | 32 | The recommended use of tetanus vaccine for trauma patients with clean and minor wounds and Complete 3-dose primary series (any tetanus-containing vaccine5) with an interval less than 5 years from the last dose |
|  | 33 | The recommended use of tetanus vaccine for trauma patients with clean and minor wounds and Complete 3-dose primary series (any tetanus-containing vaccine5) with an interval of 5 years or more from the last dose |
|  | 34 | The recommended use of tetanus vaccine for trauma patients with dirty and major wounds and Complete 3-dose primary series (any tetanus-containing vaccine) with an interval less than 5 years from the last dose |
|  | 35 | The recommended use of tetanus vaccine for trauma patients with dirty and major wounds and Complete 3-dose primary series (any tetanus-containing vaccine5) with an interval of 5 years or more from the last dose |
