# Supplementary material for: Assessing the impact of the 2018 tetanus guidelines on knowledge and practices of emergency physicians in trauma patients: a national survey study
Source: PeerJ. 2023 Sep 4;11:e16032. doi: 10.7717/peerj.16032 (PMC10484204; doi:10.7717/peerj.16032)
Supplement: Supplemental Information 3 [file peerj-11-16032-s003.docx]

破伤风预防现状调查问卷表

您好，这是一份破伤风疫苗的使用现状及影响因素的调查表。非常感谢您能抽空填写。本问卷采用匿名方式，不会记录个人信息。本问卷无对错之分，请您放心填写。衷心感谢您的帮助！

第I部分：一般信息

| 1) |  | 您所在的急诊科室 |
| --- | --- | --- |
|  | a. | 急诊内科 |
|  | b. | 急诊外科 |
|  |  |  |
| 2) |  | 您的年龄 |
|  | a. | 请输入您的年龄 |
|  |  |  |
| 3) |  | 您的性别 |
|  | a | 男 |
|  | b | 女 |
| 4) |  | 您的执业机构 |
|  | a. | 社区中心或者诊所 |
|  | b. | 二级医院 |
|  | c. | 三级医院 |
|  |  |  |
| 5) |  | 您的学历 |
|  | a. | 本科 |
|  | b. | 研究生 |
|  |  |  |
| 6） |  | **您的职称** |
|  | a | 住院医师 |
|  | b | 主治医师 |
|  | c | 副主任医师及以上 |
|  |  |  |
| 7) |  | 你所在的省份 |
|  | a. | 列表显示所有省份和地区 |
|  |  |  |
| 8) |  | 您是否接受过有关破伤风预防的培训 |
|  | a. | 是 |
|  | b. | 否 |
|  | c. | 记不清了 |

第II部分：破伤风预防相关信息

| 9) |  | 您本人近十年内是否接受过破伤风疫苗注射 |
| --- | --- | --- |
|  | a | 否 |
|  | b | 是 |
|  | c | 记不清了 |
|  |  |  |
| 10） |  | 您在过去半年内是否诊疗过创伤患者？ |
|  | a | 否 |
|  | b | 是 |
|  | c | 记不清了 |
|  |  |  |
|  |  |  |
| 11) |  | 您接诊创伤患者时询问患者的破伤风疫苗注射史吗？ |
|  | a. | 是 |
|  | b. | 否 |
|  | c. | 不清楚 |
|  |  |  |
| 12） |  | **TAT（破伤风抗毒素）或TIG（人破伤风免疫球蛋白）是** |
|  | a. | 抗原 |
|  | b. | 抗体 |
|  | c. | 不清楚 |
|  |  |  |
| 13） |  | **含破伤风疫苗（百白破疫苗，百破疫苗等）是** |
|  | a. | 抗原 |
|  | b. | 抗体 |
|  | c. | 不清楚 |
|  |  |  |
| 14) |  | 您所在的机构是否供应破伤风疫苗？ |
|  | a | 是 |
|  | b | 否 |
|  | c | 不清楚 |
|  |  |  |
| **15）** |  | **外伤后破伤风发病时间（/潜伏期）大概是多久？** |
|  | a. | 1-6天 |
|  | b. | 7-8天，一般为1~2周 |
|  | c. | 两周到一个月 |
|  | d. | 不清楚 |
| **16）** |  | **TAT（破伤风抗毒素）的半衰期是多久？** |
|  | a. | 1-4天 |
|  | b. | 5~7天，最短10~14小时 |
|  | c. | 1到2周 |
|  | d. | 不清楚 |
|  |  |  |
| **17）** |  | **未接种过破伤风疫苗的患者需要注射多少针次才能达到接近100%的保护性的抗体滴度？** |
|  | a. | 1 |
|  | b. | 2 |
|  | c. | 3 |
|  | d. | 4 |
|  | e. | 5 |
|  | f. | 不清楚 |
|  |  |  |
| **18）** |  | **外伤后实施预防破伤风措施的最佳时间为：** |
|  | a. | 24小时内 |
|  | b. | 两周内 |
|  | c. | 不清楚 |
|  |  |  |
| **19）** |  | **肠穿孔患者需要采取预防破伤风措施吗？** |
|  | a. | 不需要 |
|  | b. | 有必要 |
|  | c. | 不清楚 |
|  |  |  |
| **20）** |  | **孕妇需要采取预防破伤风措施吗？** |
|  | a. | 不需要，因为没有外伤 |
|  | b. | 有必要，根据孕妇过去的疫苗接种史决定 |
|  | c. | 不清楚 |
|  |  |  |
| **21)** |  | **儿童期已完成国家全程计划免疫的成年人需要注射破伤风疫苗吗？** |
|  | a. | 需要，每５～１０年加强１剂次，特殊情况下可每３～５年加强１剂次 |
|  | b. | 不需要，完成全程计划免疫已具备免疫力。并且继续注射破伤风疫苗并发症多，风险远大于益处 |
|  | c. | 不清楚 |
|  |  |  |
| **22)** |  | **下面关于TAT(破伤风抗毒素)或TIG(人破伤免疫球蛋白)的说法正确的是？** |
|  | a. | 可以长期预防破伤风 |
|  | b. | 只能短期（不超过一个月）预防破伤风 |
|  | c | A和b都不对 |
|  |  |  |
| **23)** |  | **您对破伤风预防的知识来源为：（多选题）** |
|  | a. | 教科书 |
|  | b. | 国际指南 |
|  | c. | 国内指南 |
|  | d. | 医院指引 |
|  | e. | 同事交流 |
|  | f. | 媒体包括报纸和网络 |
|  |  |  |
| 24) |  | **您对创伤患者使用TAT（破伤风抗毒素）预防破伤风的态度是** |
|  | a. | 强烈支持 |
|  | b. | 部分支持 |
|  | c | 强烈反对 |
|  | d | 部分反对 |
|  | e. | 无所谓 |
|  |  |  |
| 25) |  | **您对创伤患者使用人免疫球蛋白(TIG)预防破伤风的态度是** |
|  | a. | 强烈支持 |
|  | b. | 部分支持 |
|  | c. | 强烈反对 |
|  | d | 部分反对 |
|  | e | 无所谓 |
|  |  |  |
| **26)** |  | **您对创伤患者使用TAT(破伤风抗毒素)或 TIG (人破伤风免疫球蛋白)的原因是：(多选题)** |
|  | a | 因未注射TAT/ TIG发生破伤风将被认为是医疗过失，从而引起医疗纠纷和诉讼 |
|  | b | 医院同事都使用TAT（破伤风抗毒素）或 TIG (人破伤风免疫球蛋白) |
|  | c | 抗体可以立即起效，有效预防破伤风 |
|  | d | 诊疗规范要求 |
|  |  |  |
| 27) |  | **您对创伤患者使用破伤风疫苗预防破伤风的态度是：**（选a项者，无须回答28题；选b项者，须回答28题） |
|  | a. | 支持 |
|  | b. | 反对 |
|  | c. | 无所谓 |
|  |  |  |
| 28) |  | **您拒绝创伤患者使用破伤风疫苗的原因是：(多选题)** |
|  | a | 工作医院没有破伤风疫苗 |
|  | b | 疫苗预防破伤风的前提是人体需要时间产生抗体，因而不能达到有效保护作用 |
|  | c | 医院同事都不使用破伤风疫苗，都使用TAG或TIG |
|  | d | 担心疫苗的副作用如诱发自闭症，自身免疫性脑炎 |
|  | e | 患者或监护人因经费或副作用拒绝 |
|  | f | 因不注射TAG和TIG导致破伤风将被认为是医疗过失，从而引起医疗纠纷和诉讼 |
|  |  |  |
| 29） |  | **无破伤风疫苗接种史的创伤患者使用破伤风疫苗的正常用法是：** |
|  | a | 仅需注射一次百破疫苗(DT) |
|  | b | 需注射含破伤风疫苗(ＴＴ)(注：破伤风类毒素，一种疫苗制剂)，应接受全程免疫：在第０天、１个月后、７个月后分别接种一次DＴ |
|  | c | 不清楚 |
|  |  |  |
| 30） |  | **破伤风疫苗接种史不详或不足３次的创伤患者使用破伤风疫苗的用法是：** |
|  | a | 仅需注射一次百破疫苗DT |
|  | b | 需注射含破伤风疫苗(ＴＴ)，应接受全程免疫：在第０天、１个月后、７个月后分别接种一次DＴ |
|  | c | 不清楚 |
|  |  |  |
| 31) |  | 如果伤口清洁，污染不重，患者破伤风疫苗注射史不明确，您会安排患者 |
|  | a. | 消毒伤口,常规注射TAT(破伤风抗毒素），如果过敏则使用TIG (人破伤风免疫球蛋白) |
|  | b. | 消毒伤口,予以一次含破伤风疫苗(TT)，不使用TAT或人破伤风免疫球蛋白（TIG） |
|  | c. | 仅伤口处理，不使用含破伤风疫苗(TT)或TAT（破伤风抗毒素）以及人破伤风免疫球蛋白(TIG)。 |
|  | d | 消毒伤口,皮下注射TAT(破伤风抗毒素），如果过敏则使用人破伤风免疫球蛋白(TIG)并予以含破伤风疫苗(TT)注射 |
|  | e | 不清楚 |
|  |  |  |
| 32) |  | 如果伤口污染严重或开放性，患者破伤风疫苗注射史不明确，您会安排患者 |
|  | a. | 消毒伤口，必要时延期缝合。皮下注射TAT(破伤风抗毒素），如果过敏则使用人破伤风免疫球蛋白（ TIG）； |
|  | b. | 彻底清洗消毒伤口，必要时延期缝合。皮下注射破伤风抗毒素( TAT），如果过敏则使用人破伤风免疫球蛋白(TIG)；并予以含破伤风疫苗(TT)注射。 |
|  | c. | 仅彻底清洗伤口，不使用含破伤风疫苗(TT)或破伤风抗毒素（TAT）以及人破伤风免疫球蛋白(TIG)。 |
|  | d | 彻底清洗消毒伤口,并予以含破伤风疫苗(TT)注射。 |
|  | e | 不清楚 |
|  |  |  |
| 33) |  | **如果伤口清洁，污染不重，患者在全程免疫最后一次注射后的５年内受伤，您会安排患者** |
|  | a. | 清洗消毒伤口。皮下注射破伤风抗毒素（TAT），如果过敏则使用人破伤风免疫球蛋白(TIG)； |
|  | b. | 清洗消毒伤口。皮下注射破伤风抗毒素（TAT），如果过敏则使用人破伤风免疫球蛋白(TIG)；并予以破伤风疫苗(TT)注射。 |
|  | c | 清洗消毒伤口。并予以含破伤风疫苗(TT)注射。 |
|  | d. | 仅伤口处理，不使用含破伤风疫苗（TT）或破伤风抗毒素（TAT）以及人破伤风免疫球蛋白(TIG)。 |
|  | e | 不清楚 |
|  |  |  |
| 34) |  | **如果伤口清洁，污染不重，患者在全程免疫最后一次注射后的10年内（超过5年）受伤，您会安排患者** |
|  | a. | 消毒伤口。皮下注射破伤风抗毒素（TAT），如果过敏则使用人破伤风免疫球蛋白(TIG)； |
|  | b. | 消毒伤口，并予以含破伤风疫苗(TT)注射。 |
|  | c. | 仅伤口处理，不使用含破伤风疫苗（TT）或破伤风抗毒素（TAT）以及人破伤风免疫球蛋白(TIG)。 |
|  | d | 不清楚 |
|  |  |  |
| 35) |  | **如果伤口污染严重或开放性，患者在全程免疫最后一次注射后的５年内受伤，您会安排患者** |
|  | a. | 彻底清洗消毒伤口，必要时延期缝合。皮下注射破伤风抗毒素（TAT），如果过敏则使用人破伤风免疫球蛋白(TIG)； |
|  | b. | 彻底清洗消毒伤口，必要时延期缝合。并予以破伤风疫苗（TT）注射。 |
|  | c. | 仅伤口处理，不使用含破伤风疫苗（TT）或破伤风抗毒素（TAT）以及人破伤风免疫球蛋白(TIG)。 |
|  | d | 彻底清洗消毒伤口，必要时延期缝合。皮下注射TAT（破伤风抗毒素），如果过敏则使用人破伤风免疫球蛋白(TIG)并予以破伤风疫苗（TT）注射 |
|  | e | 不清楚 |
|  |  |  |
| 36) |  | **如果伤口污染严重或开放性，如患者在破伤风全程免疫后10年内（超过5年）受伤，您会安排患者** |
|  | a. | 彻底清洗消毒伤口，必要时延期缝合。皮下注射破伤风抗毒素（TAT），如果过敏则使用人破伤风免疫球蛋白(TIG)； |
|  | b. | 彻底清洗消毒伤口，必要时延期缝合。并予以破伤风疫苗（TT）注射。 |
|  | c. | 仅伤口处理，不使用含破伤风疫苗(TT)或TAT（破伤风抗毒素）以及人破伤风免疫球蛋白(TIG) |
|  | d | 彻底清洗消毒伤口，必要时延期缝合。皮下注射 TAT（破伤风抗毒素），如果过敏则使用破伤风人免疫球蛋白(TIG)并予以含破伤风疫苗（TT）注射 |
|  | e | 不清楚 |
