# Supplementary material for: Assessing the impact of the 2018 tetanus guidelines on knowledge and practices of emergency physicians in trauma patients: a national survey study
Source: PeerJ. 2023 Sep 4;11:e16032. doi: 10.7717/peerj.16032 (PMC10484204; doi:10.7717/peerj.16032)
Supplement: Supplemental Information 4 [file peerj-11-16032-s004.docx]

**Tetanus questionnaire (in English)**

Informed consent and confidentiality of interviews

Good morning/afternoon, Mr/Mrs ________. We are from “Knowledge, altitudes, and practices (*KAP) of Doctors in China Regarding Tetanus Prevention in Trauma Patients”* research group. We are working on a project concerned with Tetanus Prevention in which you could participate/participated. This study aimed to assess the KAP of doctors regarding tetanus prevention in trauma patients and to subsequently develop advocacy efforts to improve clinical doctors’ tetanus immunization training. Now, the project is just starting.

The interview will take about half an hour. All the information we obtain will remain strictly confidential, and your answers and name will not be revealed. Also, you are not obliged to answer any question you do not want to, and you may stop the interview at any time.

The objective of this study is to assess the KAP of doctors regarding tetanus prevention in trauma patients. This is not to evaluate or criticize you, so please do not feel pressured to give a specific response and do not feel shy if you do not know the answer to a question. For question 13-37, the options in red are recommended by Chinese expert consensus on tetanus immunization.

TIG: Tetanus immune globulin

DTaP: a vaccine that helps children younger than age 7 develop immunity to three deadly diseases caused by bacteria: diphtheria, tetanus, and whooping cough. It is given at the second month, fourth month, sixth month, 15^th^-18^th^ month, and at 4-6 years old.

Tdap: a booster immunization containing a lower concentration of diphtheria and pertussis toxoids than DTaP. Tdap is given at age 11. Adolescents and adults who have not yet received it should receive one dose of Tdap at the time of their next tetanus booster.

Td: a vaccine against tetanus and diphtheria only, recommended every 10 years thereafter.

Do you agree to participate in this interview?

Yes ___ No ___ If yes, continue to the next question; if no, stop the interview.

Do you have any question before we start? (Answer questions).

May I start now?

*Q1 Which department you worked in ?

1) Emergency Medicine

2) Emergency Surgery

* Q 2 How old are you (yr)？

1)20-30

2)30-40

3)40-50

4)>50

*Q3 What is your gender?

1) Male

2) Female

*Q4 What is the hospital type you worked in？

1) Primary hospital

2) Secondary hospital

3) Tertiary hospital

*Q5 what is your education level?

1) Undergraduate

2) Postgraduate

Q6 What is your Job Title ?

1. Resident
2. Attending Physician
3. Associate Chief Medical Officer or Chief Medical Officer

Q7 Which province are you from?

List of provinces for selection

*Q8 Did you receive tetanus vaccine training？

1)Yes

2)No

3)Unclear

*Q9 Did you receive tetanus boost injection in recent ten years？

1)Yes

2)No

3)Unclear

*Q10 Have you given consultations for trauma patients in the recent half year?

1)Yes

2)No

3)Unclear

*Q11 Will you query about the history of tetanus vaccination?

1)Yes

2)No

3)Unclear

*Q12 Are tetanus antitoxin or human TIG (human tetanus immunoglobulin) antibodies or antigens?

1) antigens

2) antibodies

3)Unclear

*Q13 Are tetanus-containing vaccines (DTaP, Tdap, DT, Td, or TT) antibodies or antigens?

1) antigens

2) antibodies

3)Unclear

*Q14 Does your hospital have tetanus vaccination in stock?

1)Yes

2)No

3)Unclear

*Q15 How long is the incubation period of tetanus after trauma?

1) 1-6 days

2) 7-8 days, usually 1 to 2 weeks

3）Two weeks to one month

4）Unclear

*Q16 What is the half-life of tetanus antitoxin?

1) 1-4 days

2) 5 to 7 days，may shortest as 10 to 14 hours

3)1-2 weeks

4) Unclear

Q17 How many injections do an unvaccinated patient need to get near 100% protective antibody titer?

1)1

2)2

3)3

4)4

5)5

6) Unclear

Q18 The appropriate time to implement preventive measures against tetanus after trauma is:

1) Within 24 hours

2) Within two weeks

3) Unclear

Q19 Do patients with intestinal perforation need to take preventive measures against tetanus?

1. Not necessary
2. Necessary
3. Unclear

Q 20 Do pregnant women need to take preventive measures against tetanus?

1. Not necessary as there is no trauma
2. Necessary, depending on past vaccination history of the pregnant woman
3. Unclear

Q 21 Do adults who have completed the national immunization program in childhood need to be vaccinated against tetanus?

1. Yes, boost every 5 to 10 years, or even every 3 to 5 years in specia case
2. Not necessary. Immunity is already present after completing the full schedule of immunisation. And the risks of tetanus BOOSTER are far greater than the benefits
3. Unclear

Q 22 Which of the following statements is true about TAT or TIG?

1. TAT (tetanus antitoxin) or TIG could used for long-term tetanus prevention
2. TAT (tetanus antitoxin) or TIG can only prevent tetanus for a short period of time (no more than one month)
3. Neither 1 nor 2 is correct

Q 23 The information source of tetanus prevention came from:

1. Textbook
2. International guideline
3. Chinese expert consensus or guideline
4. Hospital Guideline
5. Colleague
6. Media includes newspapers and internet

Q 24 What is your attitude toward TAT as tetanus prophylaxis measures for trauma patients ?

1. strongly agree
2. agree
3. neutral
4. disagree
5. strongly disagree

Q 25 What is your attitude to TIG as tetanus prophylaxis measures for trauma patients?

1)strongly agree

2)agree

1. neutral
2. disagree

5）strongly disagree

Q 26 The reason for you to choose TAT or TIG as tetanus prophylaxis is?

1. Concern of Malpractice suit
2. Common used measures by colleagues
3. TAT or TIG could work immediately and effectively
4. Intuitional guideline

Q 27 What is your attitude to tetanus-containing vaccines (DTaP, Tdap, DT, Td, or TT) (those who choose item 1 do not need to answer question 28; those who choose item 2 must answer question 28)

1. strongly agree
2. agree
3. neutral

4disagree

5）strongly disagree

Q 28 The reason for you not choose tetanus-containing vaccines (DTaP, Tdap, DT, Td, or TT) as tetanus prophylaxis is?

1） No vaccine available in your institution

1. Longer duration for antibody production

3) Not Commonly used measures by colleagues

4） Concern of side effect

5） Refusal by patients or Guardians due to expensive expense

6) Concern of Malpractice suit

Q 29 The recommended use of tetanus vaccine for trauma patients with no history of tetanus vaccination is:

1. Only one dose of Pertussis vaccine (DT) is required.
2. Tetanus-containing vaccine (TT) (Note: tetanus toxoid, a vaccine formulation) is required and should be fully immunized: DT on day 0, 1 month later, and 7 months later Wound management only, without tetanus vaccine (TT) or TAT (tetanus antitoxin) and human tetanus human immunoglobulin (TIG)
3. Unclear

Q 30 The recommended use of tetanus vaccine for trauma patients with incomplete 3-dose primary series with any tetanus-containing vaccine:

1） Only one dose of Pertussis vaccine (DT) is required.

2） Tetanus-containing vaccine (TT) (Note: tetanus toxoid, a vaccine formulation) is required and should be fully immunized: DT on day 0, 1 month later, and 7 months later Wound management only, without tetanus vaccine (TT) or TAT (tetanus antitoxin) and human tetanus human immunoglobulin (TIG)

3） Unclear

Q 31 The recommended use of tetanus vaccine for trauma patients with clean and minor wounds and incomplete 3-dose primary series with any tetanus-containing vaccine:

1) Thoroughly clean and disinfect the wound and postpone suturing if necessary. Subcutaneous injection of tetanus antitoxin (TAT) or, if allergic, human tetanus immunoglobulin (TIG).

2) Clean and disinfect the wound thoroughly and postpone suturing if necessary. Give tetanus vaccination (TT).

3) Wound management only, without tetanus vaccine (TT) or TAT (tetanus antitoxin) and human tetanus human immunoglobulin (TIG)

4）Clean and disinfect the wound thoroughly and postpone suturing if necessary. Subcutaneous injection of TAT (tetanus antitoxin) or, if allergic, tetanus human immunoglobulin (TIG) with tetanus vaccine (TT)

5)Unclear

Q 32 The recommended use of tetanus vaccine for trauma patients with dirty and major wounds and incomplete 3-dose primary series with any tetanus-containing vaccine:

1. Thoroughly clean and disinfect the wound and postpone suturing if necessary. Subcutaneous injection of tetanus antitoxin (TAT) or, if allergic, human tetanus immunoglobulin (TIG).
2. Clean and disinfect the wound thoroughly and postpone suturing if necessary. Give tetanus vaccination (TT).
3. Wound management only, without tetanus vaccine (TT) or TAT (tetanus antitoxin) and human tetanus human immunoglobulin (TIG)
4. Clean and disinfect the wound thoroughly and postpone suturing if necessary. Subcutaneous injection of TAT (tetanus antitoxin) or, if allergic, tetanus human immunoglobulin (TIG) with tetanus vaccine (TT)
5. Unclear

Q 33 The recommended use of tetanus vaccine for trauma patients with clean and minor wounds and Complete 3-dose primary series (any tetanus-containing vaccine5) with an interval less than 5 years from the last dose:

1. Thoroughly clean and disinfect the wound and postpone suturing if necessary. Subcutaneous injection of tetanus antitoxin (TAT) or, if allergic, human tetanus immunoglobulin (TIG).
2. Clean and disinfect the wound thoroughly and postpone suturing if necessary. Give tetanus vaccination (TT).
3. Wound management only, without tetanus vaccine (TT) or TAT (tetanus antitoxin) and human tetanus human immunoglobulin (TIG)
4. Clean and disinfect the wound thoroughly and postpone suturing if necessary. Subcutaneous injection of TAT (tetanus antitoxin) or, if allergic, tetanus human immunoglobulin (TIG) with tetanus vaccine (TT).
5. Unclear

Q 34 The recommended use of tetanus vaccine for trauma patients with clean and minor wounds and Complete 3-dose primary series (any teta-nus-containing vaccine5) with an interval of 5 years or more from the last dose:

1. Thoroughly clean and disinfect the wound and postpone suturing if necessary. Subcutaneous injection of tetanus antitoxin (TAT) or, if allergic, human tetanus immunoglobulin (TIG).
2. Clean and disinfect the wound thoroughly and postpone suturing if necessary. Give tetanus vaccination (TT).
3. Wound management only, without tetanus vaccine (TT) or TAT (tetanus antitoxin) and human tetanus human immunoglobulin (TIG)
4. Clean and disinfect the wound thoroughly and postpone suturing if necessary. Subcutaneous injection of TAT (tetanus antitoxin) or, if allergic, tetanus human immunoglobulin (TIG) with tetanus vaccine (TT)
5. Unclear

Q 35 The recommended use of tetanus vaccine for trauma patients with dirty and major wounds and Complete 3-dose primary series (any tetanus-containing vaccine) with an interval less than 5 years from the last dose

1） Thoroughly clean and disinfect the wound and postpone suturing if necessary. Subcutaneous injection of tetanus antitoxin (TAT) or, if allergic, human tetanus immunoglobulin (TIG).

2） Clean and disinfect the wound thoroughly and postpone suturing if necessary. Give tetanus vaccination (TT).

3） Wound management only, without tetanus vaccine (TT) or TAT (tetanus antitoxin) and human tetanus human immunoglobulin (TIG)

4） Clean and disinfect the wound thoroughly and postpone suturing if necessary. Subcutaneous injection of TAT (tetanus antitoxin) or, if allergic, tetanus human immunoglobulin (TIG) with tetanus vaccine (TT)

5)Unclear

Q 36 The recommended use of tetanus vaccine for trauma patients with dirty and major wounds and Complete 3-dose primary series (any teta-nus-containing vaccine5) with an interval of 5 years or more from the last dose:

1)Thoroughly clean and disinfect the wound and postpone suturing if necessary. Subcutaneous injection of tetanus antitoxin (TAT) or, if allergic, human tetanus immunoglobulin (TIG).

2)Clean and disinfect the wound thoroughly and postpone suturing if necessary. Give tetanus vaccination (TT).

3)Wound management only, without tetanus vaccine (TT) or TAT (tetanus antitoxin) and human tetanus human immunoglobulin (TIG)

4)Clean and disinfect the wound thoroughly and postpone suturing if necessary. Subcutaneous injection of TAT (tetanus antitoxin) or, if allergic, tetanus human immunoglobulin (TIG) with tetanus vaccine (TT)

5)Unclear
